# Contemporary Clinical Treatment Options and Outcomes of Aortic Valve Disease: Integrating Modern Insights into a Cohesive Therapeutic Paradigm

**DOI:** 10.3390/jcdd12060223

**Published:** 2025-06-12

**Authors:** Mladen J. Kočica

**Affiliations:** Clinic for Cardiac Surgery, UC Clinical Centre of Serbia, 8th Kosta Todorovic St., 11000 Belgrade, Serbia; kocica@sbb.rs; Tel.: +381-(69)-367-0609

Historically, the aortic valve (AV) was seen as a passive mechanical structure facilitating unidirectional blood flow by opening and closing in response to pressure changes, with little or no biological activity [1]. Modern insights have redefined the AV as a dynamic, biologically active tissue that plays significant cellular, mechanical, and immunological roles [2,3,4,5].

The valve’s ability to open and close more than 100,000 times a day is driven by mechanotransduction pathways, influencing cell behavior and matrix remodeling. The interstitial cells of the valve show remarkable plasticity, transitioning between fibroblastic, myofibroblastic, and osteoblastic phenotypes [2,4,6,7,8], contributing to both adaptive repair and pathological calcification. Moreover, the AV expresses cytokines, chemokines, and adhesion molecules, and can actively recruit immune cells, functioning as an immunologically active interface [2,4,6,7,8,9,10,11]. Dysfunction of the valve impacts left ventricular geometry, coronary perfusion, and systemic hemodynamics [12,13,14,15].

Research has increasingly emphasized that the AV should be understood from two complementary perspectives. The concept of the “aortic valve apparatus” considers the AV as an integrated anatomical and biomechanical unit—including valve leaflets, the annulus, sinuses of Valsalva, sinotubular junction, and left ventricular outflow tract—whose coordinated geometry and motion are critical for optimal valve function and effective ventriculo-arterial coupling (VAC) [16,17,18,19]. In parallel, viewing the valve as an “aortic organ” underscores its role as a biologically active tissue capable of mechanotransduction, cytokine expression, extracellular matrix remodeling, and systemic crosstalk [20]. These perspectives emphasize that successful valve interventions must preserve or restore both the mechanical integrity of the AV apparatus and the biological resilience of the aortic organ.

This reconceptualization of the AV has transformed clinical practice. The increasing application of stentless bioprostheses, AV repair, and valve-sparing procedures requires a deeper understanding of AV biomechanics and flow dynamics [13,14,15]. These advanced interventions seek not only to correct the valve but also to restore the motion of the physiologic leaflet, root elasticity, and annular–ventricular interactions to optimize global cardiovascular performance [21,22].

Importantly, both the structural and biological integrity of the AV profoundly influence VAC, a key determinant of cardiovascular performance. The mechanical dynamics of the AV apparatus modulate the afterload and flow patterns, directly impacting VAC [18,19]. Meanwhile, pathological processes at the level of the aortic organ—such as inflammation, fibrosis, and calcification—can stiffen the valve, disturb VAC, and contribute to maladaptive ventricular remodeling [18]. Modern interventions increasingly aim to restore harmonious ventriculo-arterial interaction through the preservation or correction of both the apparatus geometry and organ biology [23].

Infective aortic valve disease is also evolving. Changes in microbiology, with a rising incidence of Staphylococcus aureus and healthcare-associated infections, has altered the pathoanatomy of infective endocarditis (IE). Contemporary imaging reveals a more complex spectrum of leaflet destruction, abscess formation, and aortic root involvement, often demanding highly individualized surgical approaches [24,25].

Beyond primary AV disease, it is now well recognized that systemic conditions such as chronic kidney disease, metabolic syndrome, and autoimmune diseases significantly impact AV biology [26,27]. These systemic influences accelerate fibro-calcific remodeling and alter valve biomechanics, contributing to earlier valve dysfunction and influencing treatment outcomes. Thus, the AV serves not only as a target organ in systemic disease but also as a potential biomarker of broader cardiovascular risk [21,26].

Within this context, the management of AV disease is undergoing a remarkable transformation. Aortic valve disease remains a major global contributor to cardiovascular morbidity and mortality, particularly in aging populations [24,26,28,29]. Contemporary interventions now encompass an expanding spectrum: surgical aortic valve replacement (SAVR), transcatheter aortic valve implantation (TAVI), valve repair and sparing procedures, stentless and rapid-deployment bioprostheses, and novel pharmacologic approaches [30,31].

Clinical decision-making is now highly multidisciplinary and patient-centered, guided by heart teams and supported by advanced imaging, computational modeling, and precision medicine [24,28,29]. Future advances will increasingly rely on high-quality longitudinal data, the integration of genomic and AI-driven diagnostics, and a further elucidation of the valve’s dynamic interplay within systemic physiology [32,33].

This Special Issue of JCDD, entitled “The Evolving Understanding and Management of Aortic Valve Disease”, focuses on the contemporary achievements in clinical medicine and basic sciences that could provide new opportunities for understanding, diagnosing and treating AV disease. The nine papers featured in the First Edition of this Special Issue of JCDD, offer a multidimensional perspective on challenges and innovations in the field, collectively contributing to a more nuanced and integrated approach to care.

A comprehensive review by Notenboom and colleagues (Contribution 1) integrates insights from embryology, mechanobiology, and signalling pathways to contextualize aortic valve degeneration and therapeutic targets. The exploration of Notch, TGF-β, and second messenger systems bridges the translational gap between bench and bedside, paving the way for biologically informed interventions.

Several studies have explored the ongoing evolution of valve replacement and repair strategies. Lebehn and colleagues (Contribution 2) provide a state-of-the-art analysis of aortic regurgitation (AR) management, highlighting the application of advanced imaging techniques and biomechanical assessment to detect early myocardial fibrosis and subclinical dysfunction. This corresponds with the increasing focus on pre-emptive therapy in valve disease. The study supports earlier intervention to prevent irreversible left ventricular dysfunction and describes the evolution of transcatheter approaches, which may eventually compare with surgical techniques in certain AR patients.

Beyond procedural success, long-term ventricular function remains a decisive factor in patient prognosis. Iliuță and associates (Contribution 3) evaluate the long-term persistence of diastolic dysfunction after aortic valve replacement (AVR) in patients with aortic stenosis (AS) versus AR, highlighting differences in ventricular remodeling. AS patients showed earlier postoperative improvements in systolic and diastolic function in the left ventricle, whereas restrictive diastolic filling patterns (LVDFPs) persisted more frequently in AR patients post-AVR, which significantly impacted prognosis. This underscores the need for earlier intervention and refined patient selection based on diastolic function metrics.

Alhijab and colleagues (Contribution 4) evaluated the age-specific outcomes of mechanical versus bioprosthetic valves, reaffirming the durability of mechanical prostheses in patients under 50, while validating bioprosthetic use in older cohorts without compromising survival.

Fazmin and Ali (Contribution 5) provide a practical review of prosthesis–patient mismatch (PPM), supporting the application of proactive root enlargement when aiming to optimize hemodynamic outcomes, a principle supported by the data from multiple centers.

Toto et al. (Contribution 6) compare the mid-term clinical outcomes and hemodynamic performance of Trifecta and Perimount bioprostheses following AVR. While Trifecta valves showed lower early postoperative gradients, this advantage diminished over time, with both prostheses showing comparable mid-term outcomes in terms of major adverse cardiac and cerebrovascular events (MACCEs) or structural valve degeneration (SVD) rates. The findings underscore the importance of long-term follow-up to assess durability and performance, particularly as bioprosthetic valve use expands to younger populations.

Baric and colleagues (Contribution 7) demonstrate that aortic valve repair with external annuloplasty is feasible and durable in both bicuspid and tricuspid anatomies, finding similar survival rates (96.3% BAV vs. 97.2% TAV), no significant differences in freedom from valve reintervention, or severe AR after four years. Their results challenge the replacement-dominant mindset and emphasize repair as a viable, often underutilized alternative.

This is also the primary message of a comprehensive editorial by Djordjevic and Rudez (Contribution 8), who emphasize the need for early-career surgeons to develop expertise in AV repair through anatomical studies, advanced imaging, and mentorship in specialized centers.

Haidari and colleagues (Contribution 9) critically examine the impact of applying local antibiotics in the abscess cavity following radical debridement and patch reconstruction in patients with AV-IE. Despite theoretical benefits, their data do not support superior outcomes with antibiotic-fibrin glue compared to patch repair alone, suggesting that radical debridement remains paramount. This finding is crucial reducing the complexity of already high-risk procedures.

Collectively, these contributions underscore the transition from a binary view of surgical and transcatheter therapy to a far more layered and mechanistically informed model. Contemporary AVD management is no longer defined solely by valve gradients and procedural survival, but by the integration of structural, functional, developmental, and molecular factors that influence long-term outcomes.

The future of AVD therapy lies in personalization: tailoring interventions based not only on anatomy and the surgical risk, but also on underlying myocardial remodeling, the etiology of disease, and the molecular phenotype. As these papers demonstrate, meaningful progress comes not only from novel devices or procedures, but from the convergence of clinical rigor, surgical innovation, and basic science into a cohesive therapeutic vision.

The AV is no longer seen as a simple mechanical gate but as a complex, biologically responsive organ that is central to cardiovascular health and disease. Ongoing innovation in surgical and transcatheter therapies, coupled with a deeper understanding of valve biology and its systemic interactions, promises truly individualized care and enhanced outcomes for patients with aortic valve disease.
